# Case Report: Extra-Articular Diffuse Tenosynovial Giant Cell Tumor of the Temporomandibular Joint

**DOI:** 10.3389/fonc.2021.643635

**Published:** 2021-02-26

**Authors:** Xibiao Yang, Li Yao, Tianping Yu, Xiaoli Du, Qiang Yue

**Affiliations:** ^1^ Department of Radiology, West China Hospital, Sichuan University, Chengdu, China; ^2^ Department of Pathology, West China Hospital, Sichuan University, Chengdu, China; ^3^ Department of Radiology, Chengdu First People’s Hospital, Chengdu, China

**Keywords:** diffuse tenosynovial giant cell tumor, temporomandibular joint, computed tomography, magnetic resonance imaging, imaging features

## Abstract

Diffuse tenosynovial giant cell tumor (D-TSGCT) is a benign but locally destructive tumor of synovium that may involve joints, tendon sheaths, and bursae. Its occurrence in the temporomandibular joint (TMJ) is extremely rare. The authors reported a case of 48-year-old man with an extra-articular D-TSGCT in the TMJ with medial cranial fossa extension. computed tomography (CT) and magnetic resonance imaging (MRI) features are described. The lesion was a cystic-solid mass centered at the temporal bone without involvement of the condylar head, and its solid component presented high-density on CT and hypointensity on MRI. No signs of recurrence or metastasis was observed during 12-months of follow-up. The present report suggested the potential characteristics of radiologic imaging of D-TSGCT in TMJ.

## Introduction

Diffuse tenosynovial giant cell tumor (D-TSGCT), also referred to as pigmented villonodular synovitis (PVNS), is benign disorder arising from the synovium of the joints, bursa, and tendon sheath (1). It often involves the large joints of the extremities (2), among which knee joint is most commonly affected, followed by the hip joint and the foot joint (3). According to the anatomical site of involvement, D-TSGCT are classified as intra-articular or extra-articular, and the latter has a higher recurrence rate (4).

Although any synovium can be affected, only more than 100 cases of D-TSGCT involving the temporomandibular joint (TMJ) region have been reported, only 10 cases were classified as the extra-articular form (5–9). Due to the non-specific clinical manifestations and limited knowledge of the radiological features, D-TSGCT of TMJ is easily misdiagnosed as others tumors arising from bone and cartilage, or ear diseases which manifested as bone destruction (10). In the present study, we described a rare case of an extra-articular D-TSGCT in the right TMJ without the involvement of condylar head.

## Case Presentation

The patient was diagnosed with D-TSGCT at 48 years of age in West China Hospital. He started experiencing mild unilateral tinnitus, hearing loss from 3 years ago, the symptoms exacerbated gradually and trismus presented 6 months ago. Three months ago, the patient was diagnosed as cholesteatoma in the community hospital and no intervention was received. Soon afterwards, the patient presented to the otolaryngology clinic of our hospital. No facial numbness, facial muscle dysfunction, dysphagia, diplopia, or vision loss was reported, and the patient stated that there was no history of trauma to the face or jaw. A hard, non-tender, non-movable and poorly defined soft tissue mass in the right temporal area was revealed by physical examination. The high-resolution computed tomography (HRCT) of the temporal bone was performed first and suspected diagnosis of giant cell tumor (GCT) of the bone was made. The diffusion-enhanced Magnetic resonance imaging (MRI) was performed for further diagnosis, GCT of the bone or D-TSGCT was suspected diagnosed. The patient was then treated with surgery and the pathological examination was conducted. According to the histopathological features, in combination with radiological manifestations and clinical history, the final diagnosis of D-TSGCT was made. The timeline of diagnosis and treatment was showed in **Supplementary Figure 1**.

### Imaging Examinations

**Figure 1:**
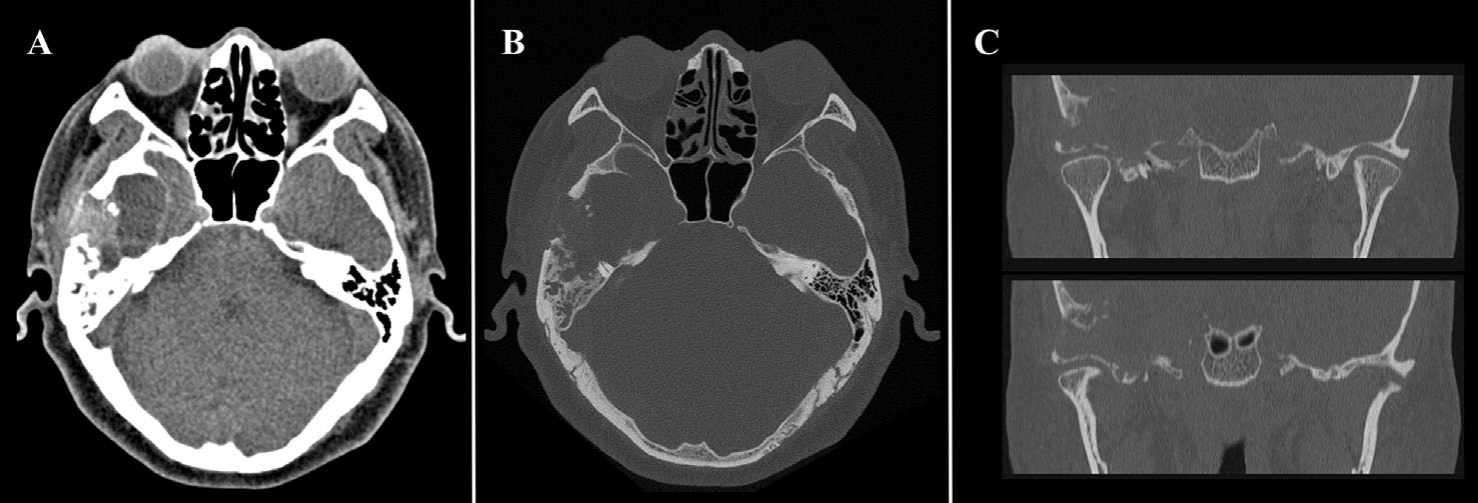
Computed tomography (CT) of a 48-year-old man with diffuse tenosynovial giant cell tumor (D-TSGCT) in right temporomandibular joint (TMJ). **(A)** A heterogeneous-density mass in the right temporomandibular joint region (soft tissue window, axial view). **(B)** Involvement of the temporal squamous and petrosal bones (bone window, axial view). **(C)** Erosion of the temporal bone while the condylar head was preserved (bone window, coronal view).

**Figure 2:**
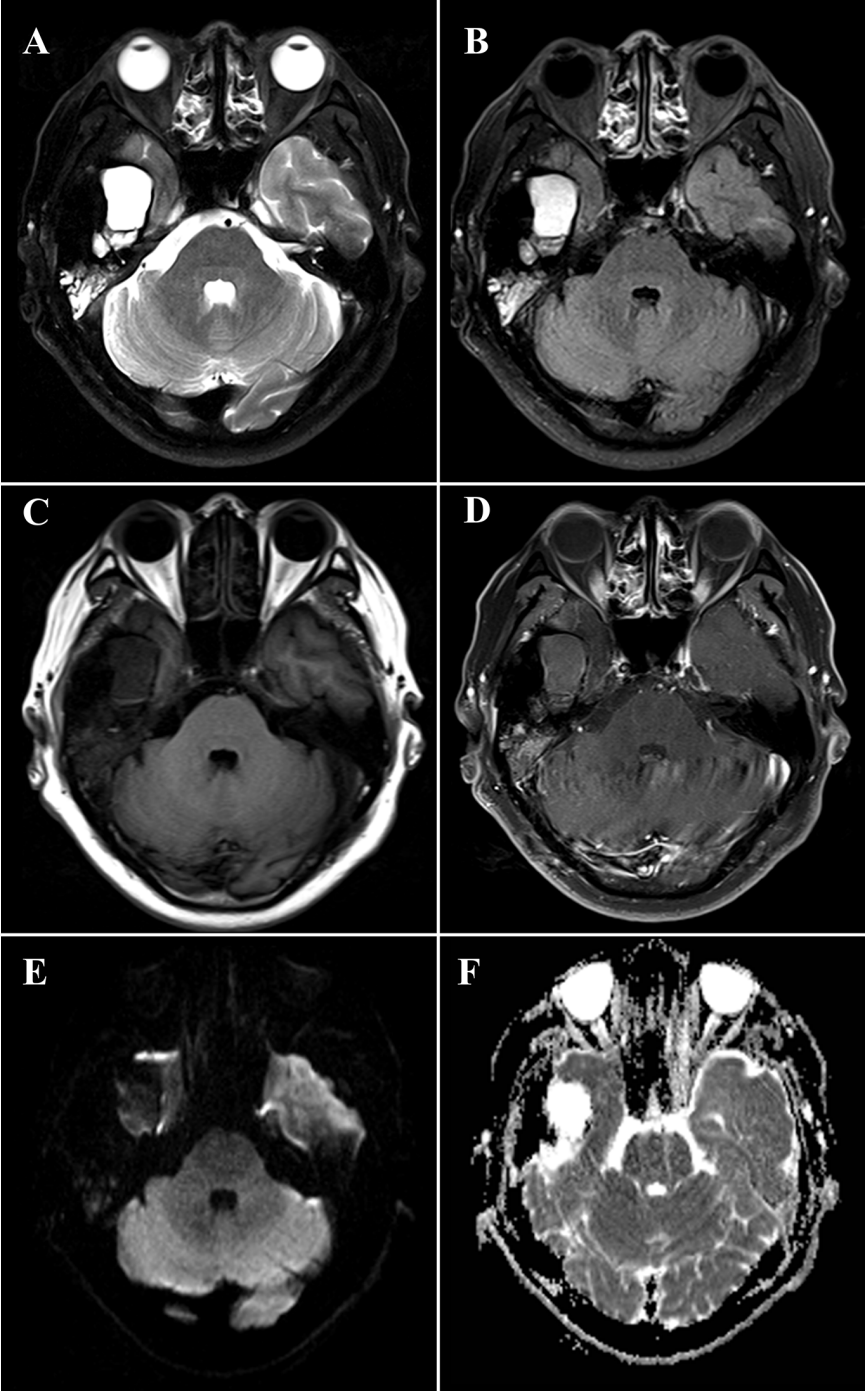
Magnetic resonance imaging (MRI) of a 48-year-old man with diffuse tenosynovial giant cell tumor (D-TSGCT) in right temporomandibular joint (TMJ). MRI reveals a predominantly hypointense lesion with focal cystic changes on T2-weight imaging **(A)**, fluid-attenuated inversion recovery (FLAIR) sequence **(B)** and T1-weighted imaging **(C)**, which infiltrates the medial cranial fossa. Mild enhancement at the wall of the cystic part and no enhancement of the solid part was observed after administration of contrast agent **(D)**. The lesion is hypointense on diffusion-weighted imaging (DWI) **(E)** and displays heterogeneous intensity on the apparent diffusion coefficient (ADC) map **(F)**.

The HRCT revealed a mixed-density mass with soft tissue density and slightly hypodensity (5.2 cm × 3.2 cm × 3.3 cm) in the right temporal squama. Incomplete bone shell was visible, and the articular surface of temporal bone was eroded while the condylar head was not involved (**Figure 1**). The fossa media was invaded through the defective temporal bone, and the temporal lobe was compressed. MRI plan scan identified an irregular and heterogeneous mass, which showed cystic components with slightly hypointensity in T1-weighted imaging (T1WI), water-like hyperintensity T2-weighted imaging (T2WI), and solid components with hypointensity both T1WI and T2WI (**Figure 2**). Hypointense signal was observed on diffusion-weighted images (DWI) (**Figure 2**). After contrast agent (Gd-DTPA) administration, slightly enhancement was present at the wall of the cystic part, no enhancement was observed in the solid part (**Figure 2**). The effusion was also observed in the adjacent mastoid.

### Surgical Findings and Pathological Examination Results

The patient underwent surgical resection on September of 2019 and the mass was completely removed. The lesion was extradural, and its texture was mixed with hard and soft component. The external auditory canal and the bone of skull base were eroded. The mass showed adhesion to the adjacent dura and adhered tightly to the surrounding tissues. The cross-section of the lesion was dark red with hard capsule.

**Figure 3:**
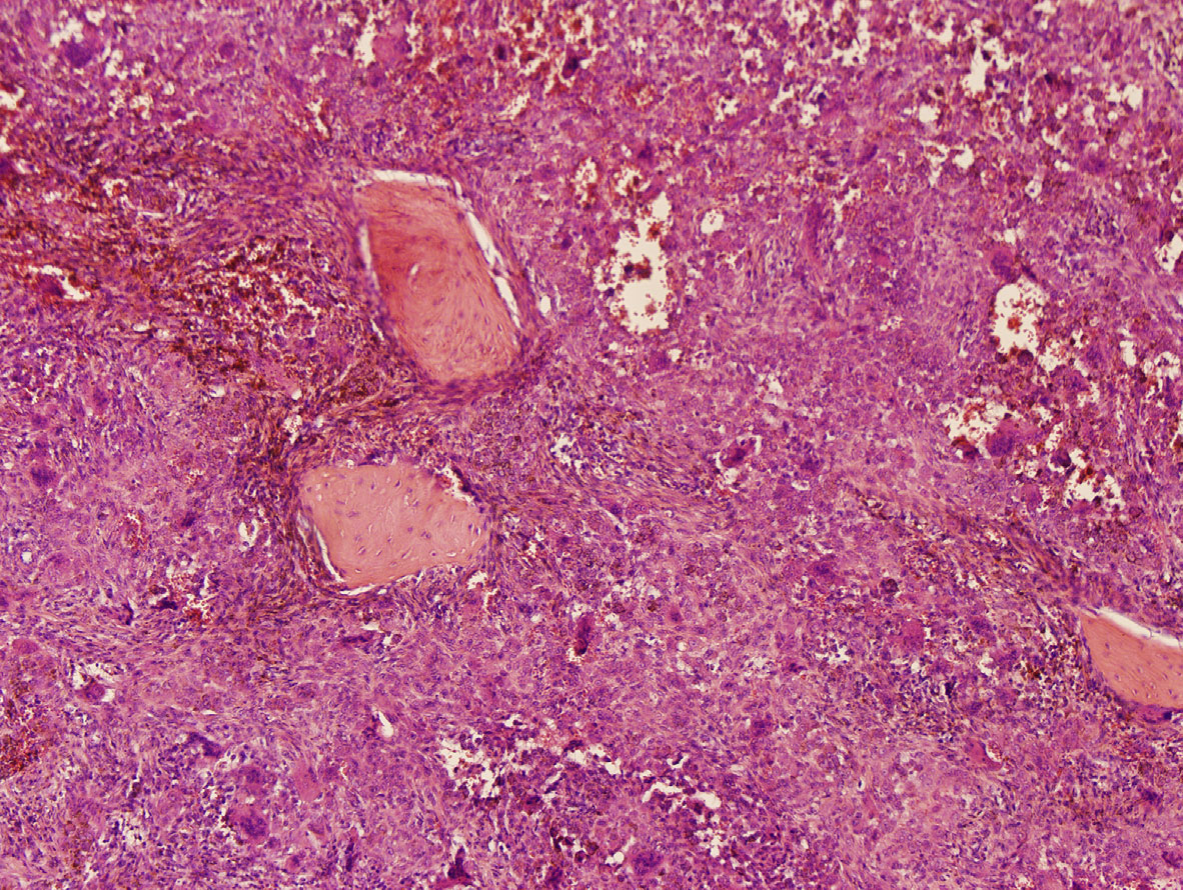
Histology of a diffuse tenosynovial giant cell tumor (D-TSGCT) of the temporomandibular joint (TMJ). Microscopic examination reveals large amount of mononuclear cells, irregularly distributed osteoclast-like giant cells, and hemosiderin pigments (hematoxylin-eosin [HE], original magnification × 100).

Microscopically, the lesion mainly composed of small, round to spindle-shaped mononuclear cells, accompanied by varying numbers of osteoclast-like giant cells, foamy cells, and hemosiderin pigments. The mononuclear cells are characterized by round or reniform nuclei, and pale cytoplasm. Osteoclast-like giant cells are usually readily apparent, which contain a variable number of nuclei. Haemosiderin deposits are virtually identified. Mitotic activity reaches up to 10 mitoses per 10 high power field (HPF). Focal necrosis and foamy cells are rarely seen. No cellular atypia was reported (**Figure 3**). A diagnosis of diffuse, extra-articular TGCT was made.

### Post-Operative Course

During 12-months of follow-up, the patient had no signs of recurrence or metastasis, the clinical symptoms were ameliorated obviously. The last time MRI data was showed in **Supplementary Figure 2**.

## Discussion

The etiology of D-TSGCT remains unclear and some possible pathogenetic theories include trauma, osteoclastic proliferation, infection, immune mechanisms, inflammation, neoplasia, and metabolic disturbances are proposed (11). The theory introduced by Jaffe is that of a reactive inflammation is most widely accepted (12), however, by the findings of clonal chromosomal aberrations (13), D-TSGCT is categorized as fibrohistiocytic tumor in the current World Health Organization (WHO) classification (14). D-TSGCT can occur at any age, but the adults aged 30-50 years are predominantly affected, with equal gender prevalence (15). D-TSGCT involving TMJ is rare, with the incidence of only 1.8 cases per one million population (16). Symptoms are nonspecific including painful or painless preauricular mass, TMJ symptoms (including click sound or trismus), and dysaudia (including hearing loss, ear fullness, and tinnitus) (17). Depression on the trigeminal and facial nerve may cause local numbness in some cases. The onset age (48 years old) and gradually aggravated dysaudia and TMJ symptoms are typical in the present case.

Macroscopically, D-TSGCT are usually large, firm or sponge-like. It has a multi-nodular appearance, with ranging in color from yellow to dark red or brown. Microscopically, most lesions are infiltrative and grow as diffuse, expansile sheets. The cellularity varies depending on the relative proportion of mononuclear cells, multinucleate giant cells, foam cells and the amount of fibrous stroma. The mononuclear component comprises larger cells and small histiocyte-like cells. Osteoclast-like giant cells, which contain a variable number of nuclei, are usually readily apparent, but may be inconspicuous in highly cellular tumors. Large amounts of haemosiderin are frequently observed in most cases and sheets of foam cells are commonly identified in the periphery of lesions. The stromal fibrosis may appear hyalinized, although it is less marked than in the localized type (18, 19).

CT and MRI provide valuable information in the diagnosis and developing treatment plan of D-TSGCT, delineating the extent of lesion as well as destruction of bone (20). Classical radiological features of the disease include high-density noncalcifying soft tissue mass on CT owing to extensive iron deposition (20). The MRI presentation of lesion varied according to the component proportions of hemosiderin, lipids, fibrous, stroma, and cellular elements (21). The lesion usually generates a low to intermediate signal in both T1WI and T2WI because of the paramagnetic effect caused by hemosiderin concentration (22). The low signal intensity caused by the presence of hemosiderin on MRI that is accentuated on gradient-recalled echo (GRE) or susceptibility-weighted imaging (SWI) sequences is also termed as the “blooming effect” (23), which is a typical feature of D-TSGCT. All these typical features are also prominent in our present case. Comparing to the lesion in the large joints which commonly involve both sides of the joint and affect joint cavity or surrounding soft tissues, the D-TSGCT in the TMJ can be centered in the bone and leads to destruction of the mandibular condyle, temporal bone, and intracranial extension (9). Cyst formation with water-like hyperintense signal at T2WI can be observed at the edge of the lesion in some cases, with non or minimal wall-like enhancement (3). Consistently, the present case manifested as heterogeneous mass centered at the temporal bone, and lateral cyst was also observed. It is worth noting that, uniquely, condylar head was preserved in this lesion.

The classification of the lesion type regarding to intra- or extra-articular can be difficult in clinical practice, because the anatomy of the TMJ region is complex (9). Wang et al. categorized the D-TSGCT involving the TMJ into 3 types on the basis of the imaging features, including bone-centered type, intraosseous type, and soft tissue type. The imaging findings of the present case were consistent with the bone-centered type, which is usually centered at the craniofacial bone around the TMJ, showing expansive bone erosion with incomplete bone shell and soft tissue mass adjacent to the bone erosion (9). The present case and previous reports (8, 9) demonstrated that, the bone-centered type lesions were relatively larger than other types which may attribute to longer illness duration. The joint surface was preserved in the early course of this type of disease, non-affected TMJ function and other mild symptoms may delayed the patient’s visit to the clinic.

The main differential diagnosis of D-TSGCT at TMJ includes GCT of the bone, synovial chondromatosis, aneurysmal bone cyst (ABCs), rheumatoid arthritis (RA), and cholesteatoma. GCT of bone characteristically involves extremities of long bones, usually manifests as expanded and soap bubble like inner structure with no calcification or mineralization. Synovial chondromatosis usually occurs in the synovium of large joints, characterized by multiple calcified nodules in the lesion area on CT. Uncalcified bodies generates hypointensity on T1WI and hyperintensity on T2WI. There is no hemosiderin deposition in the above two diseases. RA often invades multiple joints with demineralization and narrowing of the joint space. On CT or MRI, ABCs demonstrate as expansile osteolytic lesions with thin sclerotic margins, and the typical features are fluid-fluid levels with low signal intensity in the inferior layer and high signal intensity in the superior layer on T2WI. Cholesteatoma commonly affects the petrous part of temporal bone, presenting as bone destruction, accompanying with homogenous density soft tissue mass on CT, and the lesion manifests as remarkable high intensity on T2WI. Other diseases addressing preauricular swelling such as brown tumor, and synovial sarcoma are also needed to be taken into differential diagnosis. With the rare incidence, accurate diagnosis can be challenging before operation. Solid mass generating high-density on CT and hypointensity on MRI with cystic changes may be of some specificity to D-TSGCT of TMJ, in combination with clinical history and histopathological features suggest the diagnosis.

Recurrence rates of D-TSGCT after treatment varied ranging from 9 to 46%, depending on the joint affected and the length of follow-up (24, 25). The main treatment strategy of D-TSGCT is complete surgical excision of involved bones and wide synovectomy (23). However, the complete removal is challenging due to the infiltrative nature of the disease and the need to preserve the adjacent joint function. Therefore, adjuvant external beam radiation therapy (EBRT) is necessary when the surgical margin is inadequate. Additional treatments including cryosurgery, total joint arthroplasty, immunotherapy, and targeted therapy might be alternative when the complete removal is considered mutilating, but the effect of these treatments remains controversial due to the rarity of the disease and heterogeneity of the patients (26). The D-TSGCT is considered a tumor with uncertain behavior, and histologically benign D-TSGCT can rarely develop metastasis (27).

## Patient Perspective

“Four years ago, I started to find hearing loss in my right ear, the symptoms worsened gradually, and the mouth opening was restricted. I went to the community hospital first and was diagnosed as cholesteatoma. Afterwards, I went to the department of otorhinolaryngology, orthopedics and neurosurgery of West China Hospital, hoping to receive thorough treatment. After various examinations, the doctor told me that my disease might be a benign bone tumor and suggested the surgery to remove the mass. After the pathological examination, I knew that my disease is a rare tumor that occurs in the temporomandibular joint. My symptoms gradually improved after the operation and there are no signs of recurrence so far. The doctors and nurses in West China Hospital were nice, and the attention and care I received was well-organized and excellent. I do not mind my case being reported and happy that other doctors learning from that.”

## Conclusion

We describe a rare case of D-TSGCT in the TMJ without involvement of the condyle head and its CT and MRI characteristic, which is highly valuable in establishing the diagnosis.

## Data Availability Statement

The original contributions presented in the study are included in the article/**Supplementary Material**. Further inquiries can be directed to the corresponding author.

## Ethics Statement

Written informed consent was obtained from the individual(s) for the publication of any potentially identifiable images or data included in this article.

## Author Contributions

XY and LY performed the data acquisition. XY, LY, and XD performed the radiological analysis of MRI and CT images. TY performed the histopathological observation. XY, LY, and QY performed the manuscript preparation. All authors contributed to the article and approved the submitted version.

## Funding

This work was supported by the Sichuan Provincial Foundation of Science and Technology (Grant No. 2019YFS0428 and 2021YFS0077), the Foundation of the National Research Center of Geriatrics (Grant No. Z2018A07) and Science and Technology Project of the Health Planning Committee of Sichuan (19PJ078 and 19PJ247).

## Conflict of Interest

The authors declare that the research was conducted in the absence of any commercial or financial relationships that could be construed as a potential conflict of interest.

## Supplementary Material

Supplementary Figure 1 The timeline of diagnosis and treatment of the case.

Supplementary Figure 2 Magnetic resonance imaging (MRI) after 12-months follow-up, no signs of recurrence were found. A: T1-weighted imaging; B: T2-weight imaging; C: Enhanced T1-weighted imaging, axial view; D: Enhanced T1-weighted imaging, coronal view.
