# Supplementary figures and images for: Case Report: Extra-Articular Diffuse Tenosynovial Giant Cell Tumor of the Temporomandibular Joint
Source: Front Oncol. 2021 Feb 26;11:643635. doi: 10.3389/fonc.2021.643635 (PMC7953063; doi:10.3389/fonc.2021.643635)

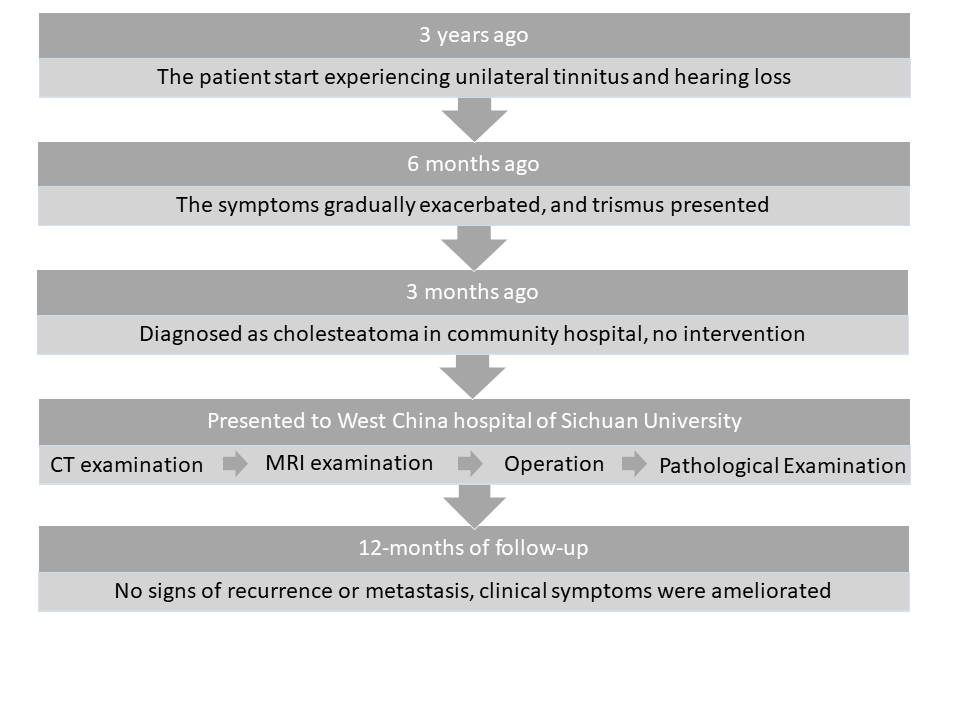

Supplement: Supplementary Figure 1 — The timeline of diagnosis and treatment of the case. [file Image_1.tif]

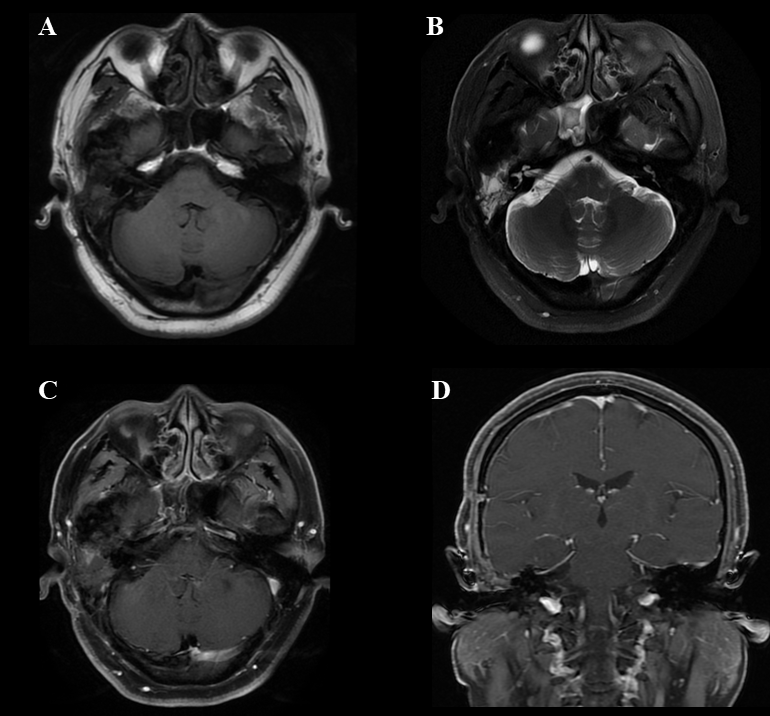

Supplement: Supplementary Figure 2 — Magnetic resonance imaging (MRI) after 12-months follow-up, no signs of recurrence were found. A: T1-weighted imaging; B: T2-weight imaging; C: Enhanced T1-weighted imaging, axial view; D: Enhanced T1-weighted imaging, coronal view. [file Image_2.tif]
